# Changes in Clinical Trials Methodology Over Time: A Systematic Review of Six Decades of Research in Psychopharmacology

**DOI:** 10.1371/journal.pone.0009479

**Published:** 2010-03-03

**Authors:** André R. Brunoni, Laura Tadini, Felipe Fregni

**Affiliations:** 1 Department and Institute of Psychiatry, University of Sao Paulo, Sao Paulo, Brazil; 2 Centro Clinico per le Neuronanotecnologie e la Neurostimolazione, Fondazione IRCCS Ospedale Maggiore Policlinico, Mangiagalli e Regina Elena, Milan, Italy; 3 Berenson-Allen Center for Noninvasive Brain Stimulation, Beth Israel Deaconess Medical Center, Harvard Medical School, Boston, Massachusetts, United States of America; Johns Hopkins Bloomberg School of Public Health, United States of America

## Abstract

**Background:**

There have been many changes in clinical trials methodology since the introduction of lithium and the beginning of the modern era of psychopharmacology in 1949. The nature and importance of these changes have not been fully addressed to date. As methodological flaws in trials can lead to false-negative or false-positive results, the objective of our study was to evaluate the impact of methodological changes in psychopharmacology clinical research over the past 60 years.

**Methodology/Principal Findings:**

We performed a systematic review from 1949 to 2009 on MEDLINE and Web of Science electronic databases, and a hand search of high impact journals on studies of seven major drugs (chlorpromazine, clozapine, risperidone, lithium, fluoxetine and lamotrigine). All controlled studies published 100 months after the first trial were included. Ninety-one studies met our inclusion criteria. We analyzed the major changes in abstract reporting, study design, participants' assessment and enrollment, methodology and statistical analysis. Our results showed that the methodology of psychiatric clinical trials changed substantially, with quality gains in abstract reporting, results reporting, and statistical methodology. Recent trials use more informed consent, periods of washout, intention-to-treat approach and parametric tests. Placebo use remains high and unchanged over time.

**Conclusions/Significance:**

Clinical trial quality of psychopharmacological studies has changed significantly in most of the aspects we analyzed. There was significant improvement in quality reporting and internal validity. These changes have increased study efficiency; however, there is room for improvement in some aspects such as rating scales, diagnostic criteria and better trial reporting. Therefore, despite the advancements observed, there are still several areas that can be improved in psychopharmacology clinical trials.

## Introduction

Clinical trials gained importance in medical research after World War II, when there was a rapid increase in drug development and research. Psychopharmacology is a field that reflects the marked increase in using clinical trials. In fact, the modern era of psychopharmacology began only in 1949, when lithium was reintroduced in psychiatry [1], being followed by the release of chlorpromazine (1954), imipramine (1958) and several others. These new drugs brought dramatic modifications in psychiatric practice and research as a new study methodology had to be developed for a field that was, until then, virtually absent from pharmacological therapies. Products of this new methodology included the development of severity rating scales and new diagnostic criteria, which eventually led to the third and fourth editions of the Diagnostic and Statistical Manual of Mental Disorders (DSM) [2]. Meanwhile, medical clinical research itself also experienced advancements such as novel study designs, better methods of blinding and randomization, more sophisticated statistical methods and better definition of outcomes [3].

Presently, psychiatric research faces important challenges. For instance, although psychiatric drugs have distinct mechanisms of action, they seem to have the same efficacy in clinical trials [4]. Moreover, the assessment of outcomes is mostly based upon severity scales that are somewhat subjective [5]. Another issue is that the diagnostic criteria are “operational”, meaning that a minimum appearance of symptoms are required to fulfill a diagnosis, which does not always reflect clinical practice [6]. Consequently, there is a concern whether psychiatric clinical trials are methodologically adequate and, if not, which aspects of trial design should be further improved [7]. Therefore, it is important to analyze the change of these aspects over time in order to understand our current methodological practice and also to be able address whether the results of past trials, which in many cases support our current therapeutics, are valid. Finally, as more recent clinical studies in psychopharmacology are failing to achieve positive results, new paths for clinical trial design are needed [7].

Therefore, a critique overview of the methodology used in past and current clinical trials can advance psychopharmacologic research. Our aim is to examine the major changes in clinical trial design by reviewing selected studies published in high-impact journals over the past sixty years. The purpose of our study is to work towards providing a better understanding on the development of psychopharmacological clinical trials, and thereby identifying future directions for its continuous advancement.

## Methods

### Eligibility Criteria

Because a review of all psychopharmacological drug clinical trials over the past sixty years is unfeasible, we reviewed only studies published in high-impact, influential general medical (The New England Journal of Medicine [NEJM], JAMA, Lancet and British Medical Journal) and psychiatric journals (Archives of General Psychiatry, The American Journal of Psychiatry [AJP], The Journal of Mental Sciences/British Journal of Psychiatry [BJP] and The Journal of Clinical Psychiatry [JCP]). It would also be unfeasible to review all of the available drugs currently and ever used in psychiatry; therefore we looked for important psychiatric drugs developed at different time periods that: (1) are currently used in psychiatry (for ease of interpretation of results); (2) are used in psychotic, mood or anxiety disorders (since such disorders rely significantly on psychopharmacological therapies) and (3) were introduced in different time periods as to cover the time period reviewed. The selected drugs were: lithium (most effective and frequently used drug for bipolar disorder) [8]; chlorpromazine (one of the most important drugs in the history of psychiatry) [9]; diazepam (the most used benzodiazepinic drug) [10]; clozapine (the most effective antipsychotic drug to date) [11]; fluoxetine (the prototypical, most studied antidepressant) [12]; risperidone (the first second-generation antipsychotic introduced) [13]; and lamotrigine (the first drug FDA approved for maintenance treatment of bipolar disorder since lithium) [14].

We also looked only for studies published within 100 months after the first retrieved article, when efficacy studies are typically conducted. The exceptions were lithium and clozapine, in which we expanded the search to twenty years, as such drugs were not initially available in the U.S. due to several deaths initially reported related to their non-monitored use [15]. Here, it should be underscored that three possible strategies were considered in our study: (1) to review all studies over 60 years on one drug only; (2) to review all studies on one mental condition only; (3) the present strategy. However, the first strategy would hinder the review of newer drugs, while older drugs are currently seldom researched for efficacy The second strategy premises diagnostic stability criteria over time, which is invalid: in 60 years, there were 4 *Diagnostic and Statistical Manual of Mental Disorders* (DSM) and 5 *International Classification of Diseases* (ICD) with different diagnostic nomenclatures. For instance, the current diagnostic of *major depressive disorder* did not exist in DSM-II in which depressed patients would probably be diagnosed as *depressive neurosis; involutional melancholia; manic-depressive illness, depressed type; or neurasthenic neurosis* [16]. Moreover, there is no single diagnosis for which different drugs were tested in efficacy trials for this entire period. Finally, the present strategy allowed us to consider several drugs and diagnoses thus extending the scope of this review examining changes over time.

### Search and Collection of the Data

**Figure 1:**
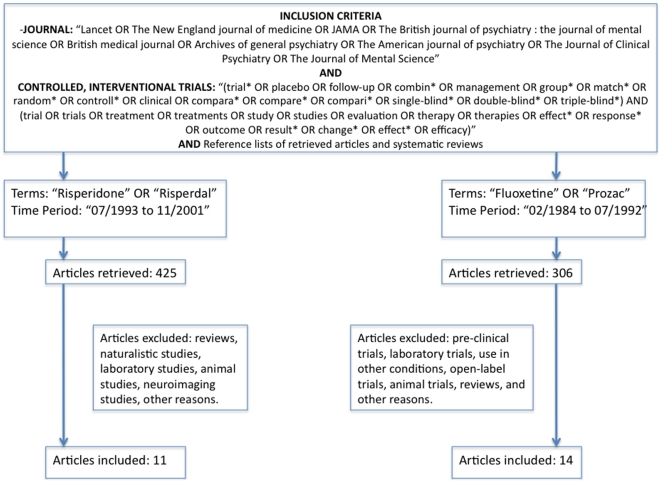
Flow chart for the selection of Risperidone and Fluoxetine studies.

**Figure 2:**
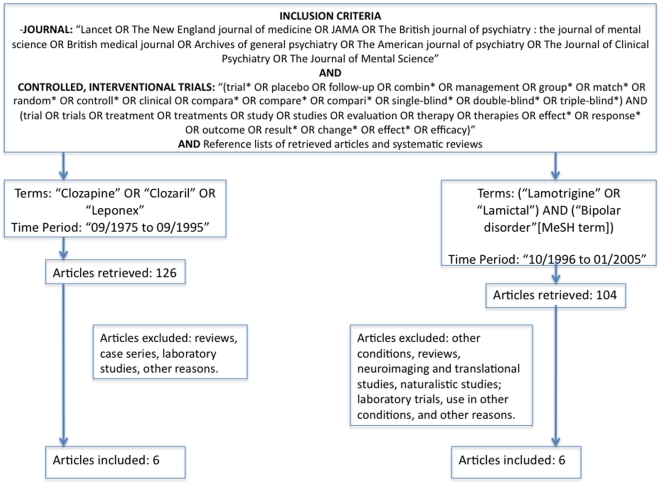
Flow chart for the selection of Clozapine and Lamotrigine studies.

**Figure 3:**
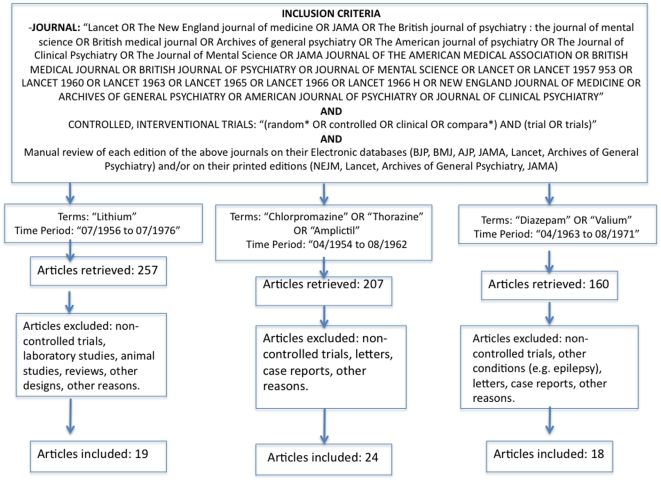
Flow chart for the selection of Chlorpromazine, Lithium and Diazepam studies.

Our search strategy is shown in Figures 1, 2 and 3. We considered the following databases: MEDLINE, Web of Science, Cochrane and EMBASE. For drugs introduced before 1970, the first author (ARB) also searched on the web sites of the journals containing past issues. The first (ARB) and the second (LT) author also performed hand search in the libraries of University of Sao Paulo Medical School and Harvard Medical School (Countway Medical Library), respectively. Finally, ARB and LT examined reference lists in systematic reviews and retrieved papers and contacted experts on the field. The keywords used for each drug review was the name of the drug, limited by the time period and by the referred journals (Figures 1, 2, 3). The procedures carried out in this review are consistent with the Cochrane guidelines for reporting systematic reviews and meta-analyses [17] and also with the QUOROM guidelines (Table S1).

The inclusion criteria for each drug were: (1) clinical studies on anxious, mood or psychotic disorders; (2) all controlled, randomized, interventional trials, whether testing either drug therapeutic or prophylactic properties (i.e., response/remission or relapse/recrudescence). We excluded: (1) other designs, such as case reports, case series, observational designs or quasi-experimental studies; (2) studies whose primary aim was not to test drug efficacy (e.g., psychometric studies); (3) clinical trials performed for other conditions than specified (e.g. lithium in hyperactive children) [18]); and (4) studies in animals. Since all selected journals are published in English, language restriction was not an issue.

### Data Extraction

The first author (ARB) performed the data extraction and compiled the variables extracted to the database, while the second author (LT) checked if data were correctly recorded. The third author (FF) reviewed a random sample of the articles to recheck for errors in data extraction or interpretation. Disagreements were resolved by consensus. We designed a semi-structured checklist, based on previous methodological reviews of clinical trials [19], [20], [21], [22], [23] to address the following aspects:

1. *general characteristics* (author names, publication year, journal published and sources of financial support);
2. *abstract reporting*, in which the complete report of background, methods and results in the abstract (yes/no for each one) were considered;
3. *study design*, assessing number of centers (uni- *vs.* multicentric), use of washout (yes *vs.* no *vs.* drug-free), use of placebo arm (yes *vs.* no), study design (2-arm *vs.* 3-arm *vs.* other designs), use of intention-to-treat analysis (yes *vs.* no);
4. *participants section*, assessing the sample size, the reporting of informed consent (yes *vs.* no) and eligibility criteria (clear *vs.* unclear), the method for evaluating diagnostic severity (personal judgment *vs.* rating scales) and for confirming the diagnostic (clinical interview *vs.* structured questionnaires);
5. *methods section*, assessing whether the method of randomization reported was adequate *vs.* inadequate *vs.* biased; the method for allocation concealment (adequate *vs.* inadequate *vs.* biased); sample size calculation reporting (yes *vs.* no); and statement of primary hypothesis (adequate *vs.* inadequate);
6. *results reporting*, assessing the reporting of baseline comparisons (adequate *vs.* inadequate), of adverse effects (adequate *vs.* inadequate) and of dropout reasons (adequate *vs.* inadequate); and the use of parametric tests (yes/no).
7. *conclusion section*, assessing whether the trial was reported as positive *vs.* negative *vs.* unclear; and whether the conclusions presented were consistent with the results (consistent *vs.* inconsistent *vs.* dubious).

The criteria used for data classification are presented in Table 1.

**Table 1:** Criteria used for data classification in the present review.

Abstract Reporting	*Background*	Adequate - when a synthesis of the current knowledge and study objectives was provided.
	*Methods*	Adequate - when the trial design, the subjects, and the interventions were described.
	*Results*	Adequate - when the results, the primary outcome and the main conclusions were described.
Study design	*Wash-out*	Yes - if prior treatments were withdrawn before the trial started.
	*Intention to treat*	Yes - if the analysis considered the entire sample, before dropouts.
	*Sample Size Calculation*	Yes - if an analysis for sample size was performed and presented.
	*Informed consent*	Yes - if the use of an informed consent is described.
Subjects	*Eligibility criteria*	Clear - the study population can be reproducible with the information given.
		Unclear - The study population cannot be reproducible and/or there is evidence of enrollment bias.
	*Diagnostic Criteria*	Clinical interview - the diagnostic was confirmed by a clinical interview.
		Structured form - the diagnostic was confirmed by using an structured questionnaire.
	*Diagnostic Severity*	Rating scales - when rating scales were used to assess severity.
		Physician judgment - when the physician judged the degree of improvement and/or severity.
Methods	*Randomization*	Adequate - when the method of sequence generation was reported.
		Inadequate - when the sequence generation method was not reported.
		Evidence of bias - when the method was described but it was biased.
	*Allocation*	Adequate - when the method of allocation concealment was reported.
		Inadequate - when the allocation concealment method was not reported.
		Evidence of bias - when the method was described but it was biased.
	*Primary Hypothesis*	Adequate - the primary hypothesis was clearly stated.
		Inadequate - the primary hypothesis was not or was incompletely stated.
Results	*Baseline Comparisons*	Adequate - when the groups were compared at baseline.
		Inadequate - when the groups were not compared at baseline.
	*Adverse Effects*	Adequate - the adverse effects were fully reported.
		Inadequate - the adverse effects were not or were partially reported.
	*Dropout reasons*	Adequate - the reasons of dropouts were assessed and presented.
		Inadequate - the dropout reasons were not presented or not fully reported.
	*p value*	Adequate - the p value of the primary outcome was reported.
Conclusion	*Trial result*	Positive - the authors stated their main hypothesis was proved.
		Negative - the authors stated they failed to prove their main hypothesis.
		Unclear - the authors does not clear state whether or not their main hypothesis was proved.
	*Consistency*	Yes - the conclusion is supported by the study results.
		Dubious - lack of trial quality or overinterpretation of results.
		No - There is clear evidence of bias in the study or the conclusion is clearly not coherent with the results shown.

### Data Analysis

The variables collected were managed as outcome variables and each one was analyzed separately. “Year” was the main predictor variable as to assess whether the outcome changed over time. We performed a separate analysis using drug class (3 levels: antipsychotics – clozapine, chlorpromazine and risperidone; mood stabilizers – lamotrigine and lithium; and others – fluoxetine and diazepam) as to assess a possible drug class confounding effect. “Year” was treated as a continuous and an ordinal variable (divided in equal quartiles). When treated as continuous, logistic regressions were applied; when ordinal, we used the chi-square or the Fisher's exact test. Analyses were performed using Stata statistical software, version 9.0 (StataCorp, College Station, TX, USA) and SPSS Software, version 16. As shown below, analyses using both methods yielded quite similar results.

## Results

Ninety-one articles were reviewed, 24 (26.7%) on chlorpromazine, 20 (21%) on lithium, 8 (8.9%) on diazepam, 6 (6.7%) on clozapine and lamotrigine each, 16 (17.8%) on fluoxetine and 11 (12.2%) on risperidone. Most trials were published in the BJP (30 trials, 33%), the JCP (20 trials, 22%) and the AJP (19 trials, 21%). We did not identify any trials from NEJM. Twenty- four trials were performed in 1961 or earlier, 23 trials throughout 1962–74, 22 trials throughout 1975–89 and 22 trials from 1990 to 2003. Also, we were not able to identify the major source of sponsorship in 48 (52%) of the studies. In 36 studies, we classified the sponsorship as public while in 7 the classification was considered private. The issue here is that newer trials have many authors and each one usually has one or more funding source. For example, one article [24] reported funding from a NIH grant, two foundations award grants, and a public, local mental health grant. The first author was a member of the speaker's bureau for four pharmaceutical companies, one of them being the sponsor of the tested drug. In such cases, we classified the sponsorship as “unclear”. As this issue occurred in 52% of the studies, we did not perform further statistical analyses.

The individual characteristics of each trial are presented in the Appendix (Table S2). Table 2 presents the summary characteristics of the reviewed studies. Table 3 shows the analyses run for categorical variables.

**Table 2:** Shows the summary characteristics of the studies.

Time Period		1961 and earlier	1962–1974	1975–1989	1990 and after
*General characteristics*					
Number of trials		24	23	22	22
Drug class	Antipsychotic	24	0	4	13
	Mood Stabilizer	0	15	5	6
	Other	0	8	13	3
Disorder	Psychosis (*)	23	0	3	12
	Affective disorders (**)	0	16	4	6
	Anxiety disorders	1	7	0	2
	Unipolar depression	0	0	15	2
Centers	Multicentric	3	8	6	15
Number of subjects	Mean (SE)	120 (30)	69 (14)	86 (15)	214 (65)
*Abstract*					
Background	Adequate	2	2	8	17
Methods	Adequate	8	14	14	21
Results	Adequate	5	5	8	21
*Study design*					
Wash-out	Reported	4	2	12	17
Intention-to-treat	Performed	0	1	4	16
Sample size calculation	Reported	0	1	0	7
Informed Consent	Reported	0	3	15	19
Number of arms	2-arm	6	14	20	18
	3-arm	4	2	2	2
*Subjects*					
Eligibility criteria	Clear	2	2	18	22
Diagnostic criteria	Structured form	0	1	0	9
Diagnostic severity	Rating scales	12	13	20	19
*Methods*					
Randomization	Adequate	4	2	0	11
Allocation	Adequate	5	0	1	3
Primary hypothesis	Adequate	0	4	2	12
*Results*					
Baseline comparisons	Adequate	4	11	11	21
Adverse effects	Adequate	5	4	11	16
Dropout reasons	Adequate	15	14	15	19
p value	Adequate	6	7	10	18
*Conclusions*					
Trial result	Positive	7	15	12	13
	Negative	10	6	8	6
	Unclear	7	2	1	3
Consistency	Yes	1	11	13	18
	Dubious	14	10	7	4
	No	9	2	1	0

All data are presented as the number (count) of trials per period, except the number of subjects, which is presented as mean and standard error.

(*)includes Schizophrenia, “Paraphrenia”, “elderly patients with psychosis” and other types of non-affective psychosis.

(**)includes Maniac-Depressive Illness, Mania, and Bipolar Disorder.

**Table 3:** Data analysis and study results.

Outcome variables		Predictor variables					
		Year (continuous)		Year (ordinal)		Drug Class	
*Abstract reporting*	*Level*	B (S.E.)	p	χ2 or ANOVA	p	χ2 or ANOVA	p
*Background*	*Adequate (vs. Inadequate)*	−0.11(0.02)	<0.01	33.8	<0.01	2.54	0.28
*Methods*	*Adequate (vs. Inadequate)*	−0.08 (0.02)	<0.01	19.4	<0.01	5.4	0.07
*Results*	*Adequate (vs. Inadequate)*	−0.1 (0.02)	<0.01	37.1	<0.01	1.6	0.45
*Subjects section*			p				
*Eligibility Criteria*	*Clear (vs. Unclear)*	−0.23 (0.05)	<0.01	35.4	<0.01	4.03	0.13
*Diagnostic Criteria*	*Interview (vs. Structured)*	0.15 (0.5)	<0.01	17.4	<0.01	5.7	0.06
*Diagnostic Severity*	*Scales (vs. Judgment)*	0.06 (0.02)	<0.01	66.3	<0.01	4.56	0.11
*Informed Consent*	*Yes (vs. No)*	0.17 (0.03)	<0.01	49.7	<0.01	12.45	0.01
*Sample Size Estimation*	*Yes (vs. No)*	0.14 (0.05)	<0.01	19.6	<0.01	3.77	0.15
*Number of Subjects* (*)		2.58 (1.24)	0.04	3.06	0.03(*)	1.83	0.17
*Study Design*							
*Number of Arms*	*2 (vs. 3 and others)*	−0.08(0.02)	<0.01	25.8	<0.01	5.08	0.08
*Use of Placebo*	*Yes (vs. No)*	0.02(0.01)	0.13	5.7	0.13	9.8	0.01
*Wash-out period*	*Yes (vs. No)*	0.05 (0.02)	<0.01	39.96	<0.01	6.32	0.17
*Centers*	*Uni (vs. Multicentric)*	0.06 (0.02)	<0.01	16.53	<0.01	1.26	0.53
*Intention-to-Treat*	*Yes (vs. No)*	0.15 (0.03)	<0.01	42.6	<0.01	0.56	0.75
*Methods section*							
*Randomization*	*Adequate (vs. Inadequate)*	0.05 (0.02)	0.01	20.83	<0.01	8.78	0.02
*Allocation*	*Adequate (vs. Inadequate)*	−0.02 (0.02)	0.39	6.8	0.08	7.96	0.02
*Primary Hypothesis*	*Adequate (vs. Inadequate)*	−1.4 (0.26)	<0.01	24.34	<0.01	5.78	0.55
*Results reporting*							
*Baseline comparisons*	*Adequate (vs. Inadequate)*	0.08 (0.02)	<0.01	28.82	<0.01	6.72	0.04
*Adverse Effects*	*Adequate (vs. Inadequate)*	0.07 (0.02)	<0.01	19.37	<0.01	2.91	0.23
*Reasons for drop-outs*	*Adequate (vs. Inadequate)*	0.09 (0.03)	0.34	6.1	0.41	5.63	0.21
*p value*	*Adequate (vs. Inadequate)*	0.11 (0.03)	<0.01	32.1	<0.01	13.7	0.08
*Test used*	*Para (vs. Non-para)*	0.05 (0.2)	<0.01	15.06	<0.01	5.57	0.06
*Conclusion section*							
*Trial result*	*Positive (vs. others)*	−0.02 (0.01)	0.16	10.23	0.11	14.3	0.06
*Consistency*	*Yes (vs. others)*	−0.01 (0.2)	<0.01	35.8	<0.01	19.2	<0.01

We used the logistic regression model to analyze the association between each outcome variable (treated as categorical data) and the predictor variable year (treated as continuous data). Also, we used the Chi-square test or the Fisher's exact test for the predictor variables year (when treated as ordinal data, divided in quartiles) and Drug Class, treated as ordinal data, divided in mood stabilizers, antipsychotics and others (fluoxetine and diazepam).

(*)for number of subjects we used the one-way ANOVA. B (SE) represents B value and its standard deviation.

**Figure 4:**
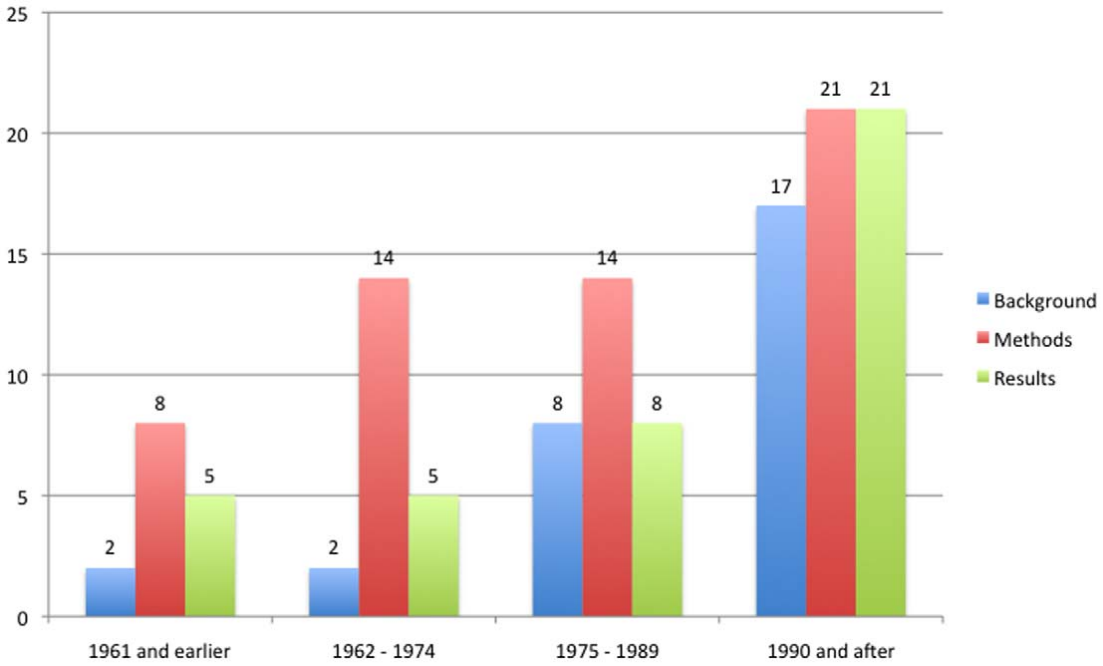
Changes in abstract reporting over time. Blue, red, and green bars show the number of trials adequately reporting background, methods and results in the abstract, respectively, at each period of time. The number of trials per period was 24 (1961 and earlier), 23 (1962–1974), 22 (1975–1989) and 22 (1990 and after).

Regarding abstract reporting, there was an improvement in quality reporting in all sections of an abstract (background, methods and results) over time (p<0.01 for all analyses) (Figure 4).

In the “participants” section, we noticed a significant improvement in clear eligibility criteria (p<0.01). Examples of unclear eligibility criteria were: “anxiety enough to require a tranquilizer” (comparing Diazepam and Lorazepam) [25]; “the most aggressive and disturbed untreated patients” (comparing Chlorpromazine and Prochlorpromazine)[26]; “patients needing ECT” (comparing Diazepam and Amitryptyline) [27]; and “when chlorpromazine was [considered] the treatment of choice” (comparing Chlorpromazine and ‘Pacatal’). Also, newer trials used more structured interviews to confirm a diagnosis, while older trials relied mainly on clinical interviews (p<0.01). Accordingly, newer trials used severity rating scales more frequently than older trials, which assessed severity based on physician's judgment (p<0.01). A performance bias was also possible as the raters were not blinded to the interventions what could theoretically favors the experimental arm in some of the studies. It was also noticed that newer trials performed or reported more sample size calculations than older trials (p<0.01). The sample sizes of newer studies were marginally larger (p = 0.04 and 0.03 for year as continuous and as ordinal, respectively) than older studies; however this difference could be explained by a recent (1995) trial [28] that is twice as large as compared to next largest study [29]. Finally, newer trials reported or used more informed consents than the older trials (p<0.01). Signs of poor ethical standards were observed in some of the older trials. For example, in one relapse trial of lithium vs. placebo for maniac-depressive illness, ambulatory patients had their drug changed to placebo without knowing [30].

**Figure 5:**
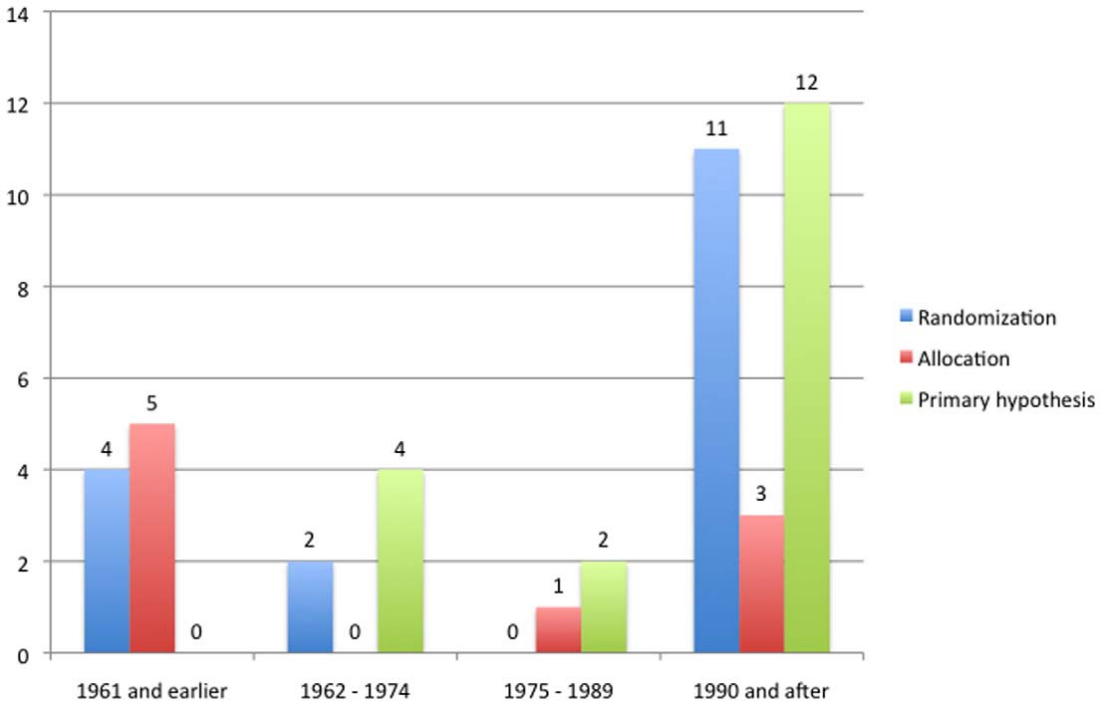
Changes in study design over time. Blue bars represent the number of trials performing two-arm studies; red bars are the trials performing three-arm studies. Green bar represent studies using other designs.The number of trials per period was 24 (1961 and earlier), 23 (1962–1974), 22 (1975–1989) and 22 (1990 and after).

**Figure 6:**
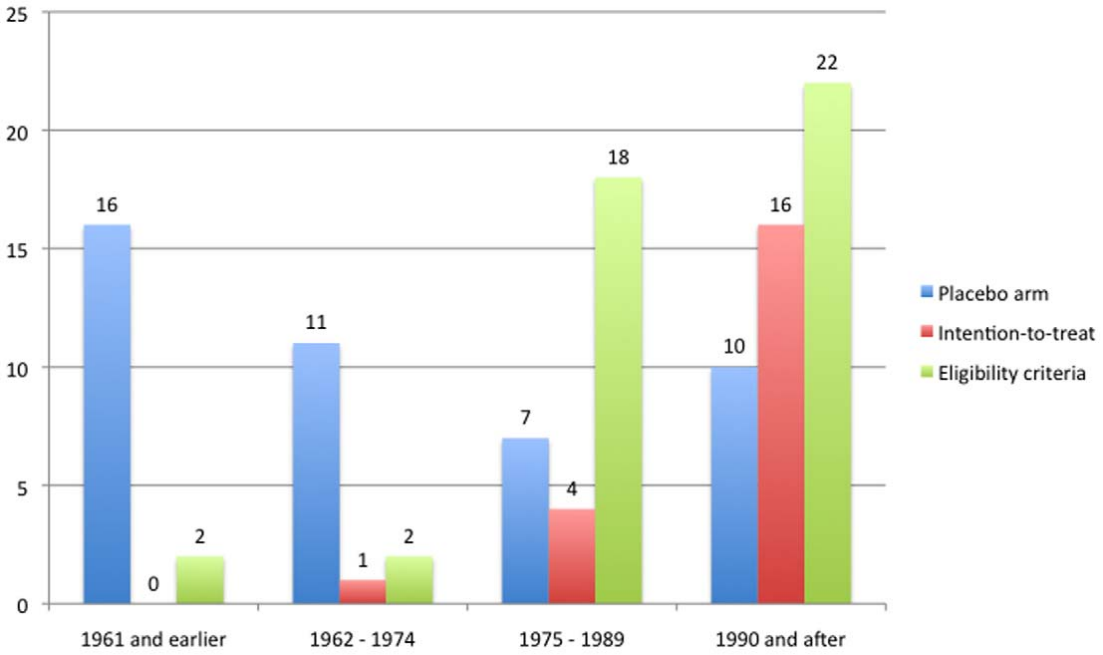
Changes in study methodology over time (1). Blue bars represent the number of trials that had a placebo arm at each period of time. Red bars represent the number of studies using intention-to-treat techniques. Green bars represent the number of studies that clearly reported their eligibility criteria.The number of trials per period was 24 (1961 and earlier), 23 (1962–1974), 22 (1975–1989) and 22 (1990 and after).

Regarding study design, a two-arm, parallel design was most often used in newer trials, when compared to the three-arm and other designs (p<0.01) (Figure 5). The number of studies using placebo arms did not change over time (p = 0.13 for year as continuous and ordinal). Newer studies were also associated with multicentric designs, drug washout prior to the trial onset, and intention-to-treat analyses (p<0.01for all variables) (Figure 6).

**Figure 7:**
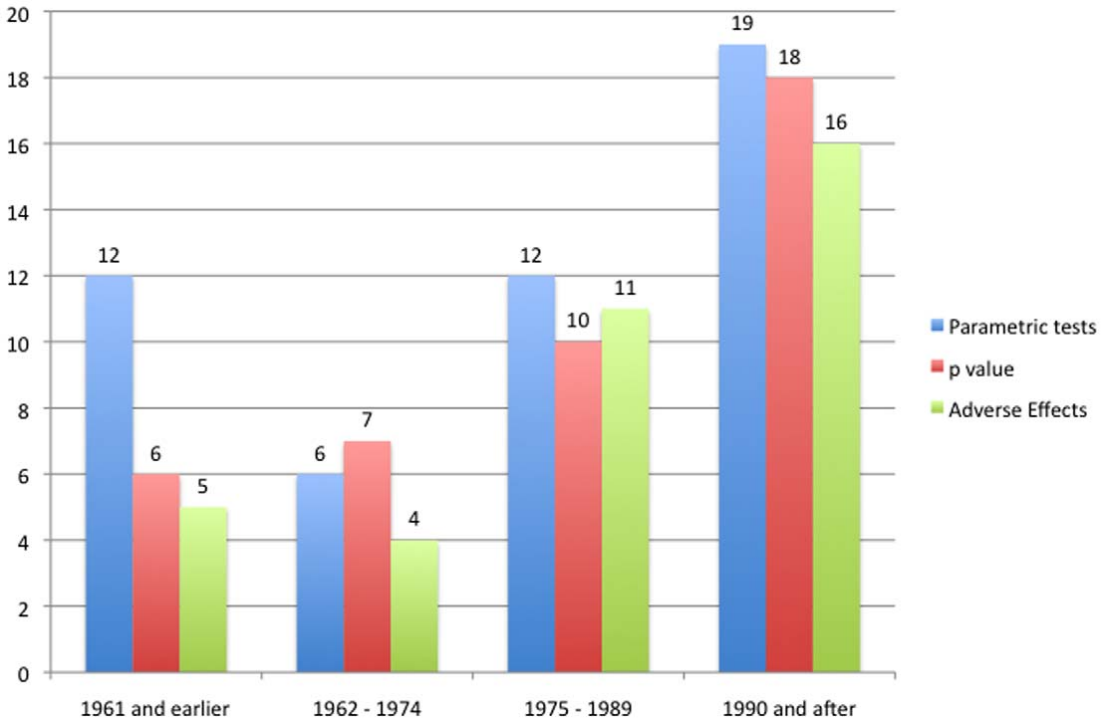
Changes in study methodology over time (2). Blue bars represent the number of trials that adequately reported randomization methods at each period of time. Red bars represent the number of studies adequately reporting allocation methods. Green bars represent the studies that adequately stated their primary hypothesis.The number of trials per period was 24 (1961 and earlier), 23 (1962–1974), 22 (1975–1989) and 22 (1990 and after).

We noticed that six studies reported clearly biased methods of randomization and allocation: alternated admission in the ward [31], using 25 red and 25 black cards for group assignment [32], physician's judgment on the best therapy (insulin coma or chlorpromazine) [33]; randomization and assignment performed by the hospital pharmacist, “the *choice* having been made by him *at random*”, although 45 patients received active drugs and 25 control tablets [34]; assignment according to the patient willingness to do weekly blood tests (mandatory when taking clozapine) [35]; and physician's judgment on the best therapy (olanzapine or risperidone) [36]. In these cases, although the methods were reported, we considered them as “inadequate” and were analyzed accordingly. The results showed that the reporting of sequence generation methods improved over time (p = 0.01 and p<0.01 for year as continuous and as ordinal, respectively) while the allocation concealment did not (p = 0.39 and p = 0.08 for year as continuous and as ordinal, respectively). However, the overall number of trials reporting the randomization and allocation methods was low (18% and 10%, respectively). Also, eight trials were not double-blinded or single-blinded with external raters, four of them compared patients using pharmacological *vs.* non-pharmacological treatments (ECT, insulin therapy or psychotherapy) [31], [33], [37], [38]. One used a no-treatment arm [39], one was initially double-blinded but patients and physicians discovered the assignment because the pills taken differed in color, size and quantity for each arm [32], one had patients in one group doing weekly blood tests while the other group did not [35]; and in another study, patients knew their assignment groups [36]. The other 83 trials used double-blinded or “double-dummy” techniques. Figure 7 visually assesses these changes.

Regarding the results section, newer trials adequately reported more than older trials: “baseline group comparisons” (p<0.01), “adverse effects” of drugs (p<0.01) but not “reasons for drop-outs” (p = 0.34 and p = 0.41 for year as continuous and ordinal, respectively). Also, newer trials reported more than older trials the *p* statistics (p<0.01) and used more parametric tests (p<0.01).

In the conclusion section we assessed whether the results were presented as positive, negative or did not provide a clear statement. We also recorded whether or not the conclusion is supported by the results; accordingly to our previous definitions (Table 1). Some examples of the 35 trials classified as “dubious” were: a lamotrigine vs. placebo trial that concluded the active drug “is associated with superior efficacy” although this was true for some but not all analyses [40]; and a trial comparing acetophenazine vs. diazepam in anxious depression that reported several comparisons and was not able to conclude which one was better [41]. Examples of inconsistent conclusions were: a underpowered trial that compared lithium vs. chlorpromazine in 23 patients with mania that concluded that “lithium is apparently superior (…) in mania”. Although the author reported that “lithium was superior on all scales, this was not statistically significant on any(…)”. He explained his conclusion arguing that “in this study and all previous ones these findings are based on poor methodological techniques…. due to the nature of the illness and the [nature of] the drugs, no reasonable (…) trial can ever be performed” [42]; and a 1959 trial in which the author compared the effects of 4 drugs in geriatric patients with various diagnostics – his severity assessment was based on four dimensions (social, intellectual, mood and thought improvement) and included his clinical evaluation, a psychologist evaluation and the “nurses and psychiatric aides” evaluation performed two times a week for 18 weeks. At the end, though, the author stated that “since it was impossible quantitatively to weigh these fluctuating factors, the final judgment in assessing the patient's responses was necessarily a clinical decision based on the accumulated data” [43]. Importantly, the 12 studies rated as “inconsistent” had some signs of methodological flaws. Four were single-blinded, 4 did not report the gender proportion, 3 did not report the mean age of the subjects, none used intention-to-treat, 10 had unclear eligibility criteria, 9 did not report randomization methods and 10 did not state detailed adverse effects.

**Figure 8:**
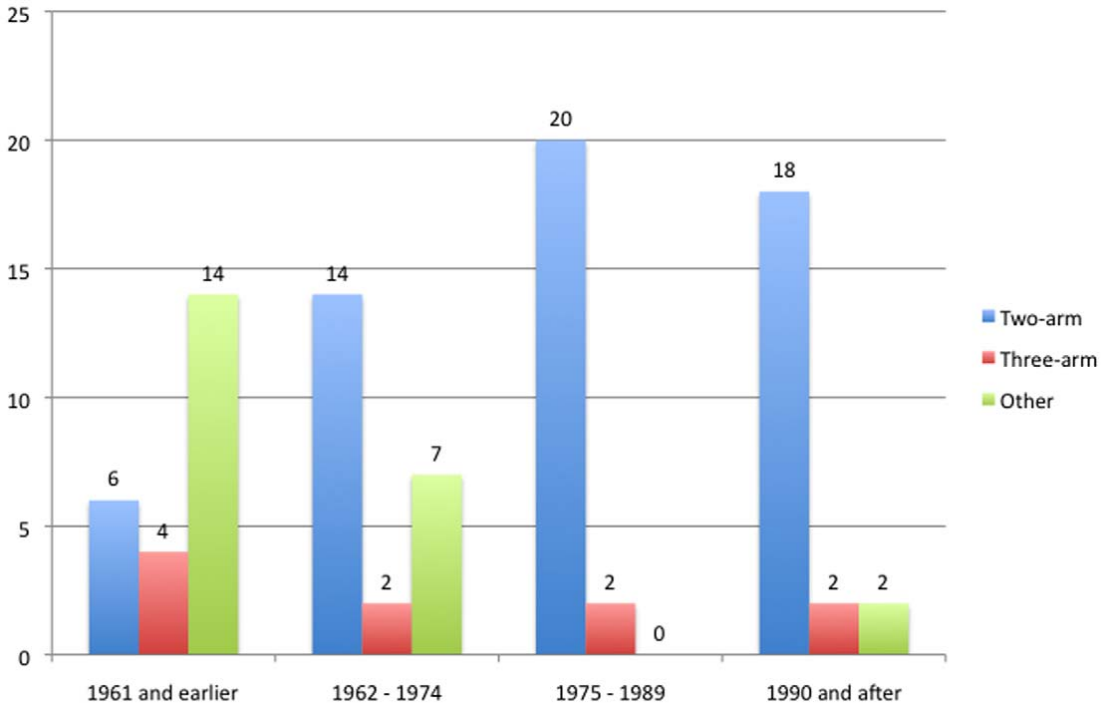
Changes in results reporting over time. Blue bars represent the number of studies that applied parametric tests in their primary outcome at each time period. Red bars represent the number of studies reporting *p* values at each time period. Green bars represent the number of studies fully reporting adverse effects at each time period.The number of trials per period was 24 (1961 and earlier), 23 (1962–1974), 22 (1975–1989) and 22 (1990 and after).

**Figure 9:**
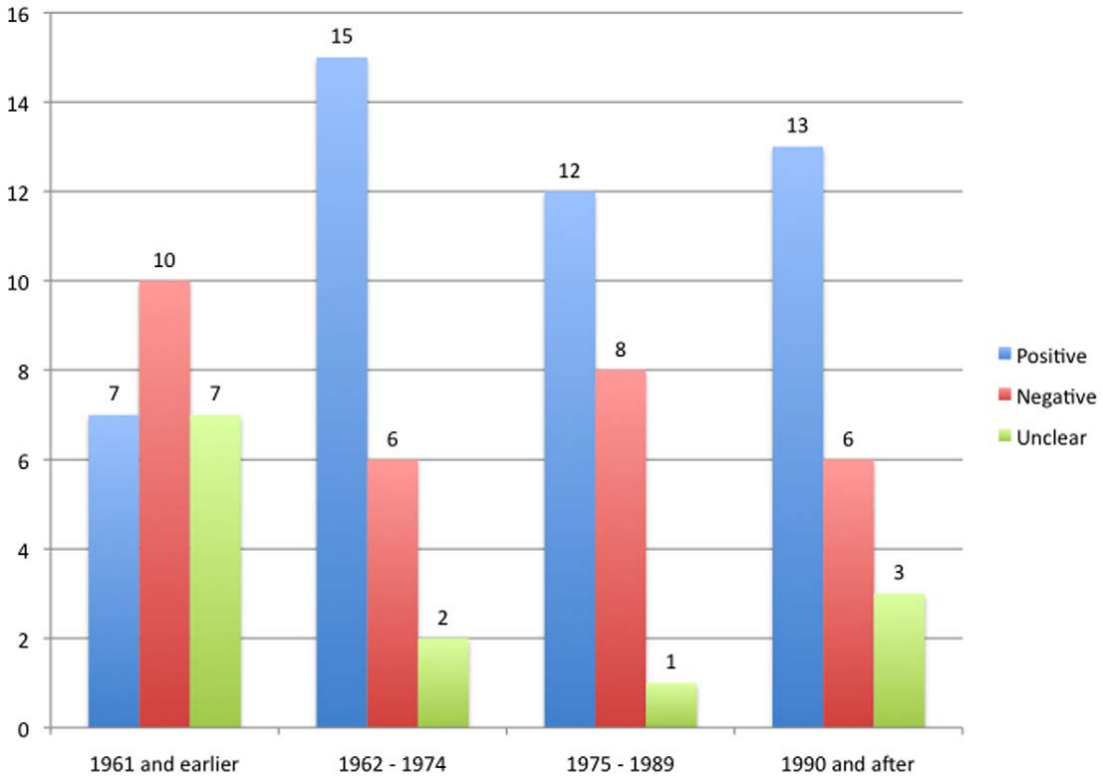
Study outcomes over time. The figure shows the number of studies in which the conclusion was positive (i.e., confirmed the primary hypothesis) (blue bars), negative (did not confirm the primary hypothesis) (red bars) or unclear, when the authors did not present a clear conclusion/interpretation of their results (green bars).The number of trials per period was 24 (1961 and earlier), 23 (1962–1974), 22 (1975–1989) and 22 (1990 and after).

**Figure 10:**
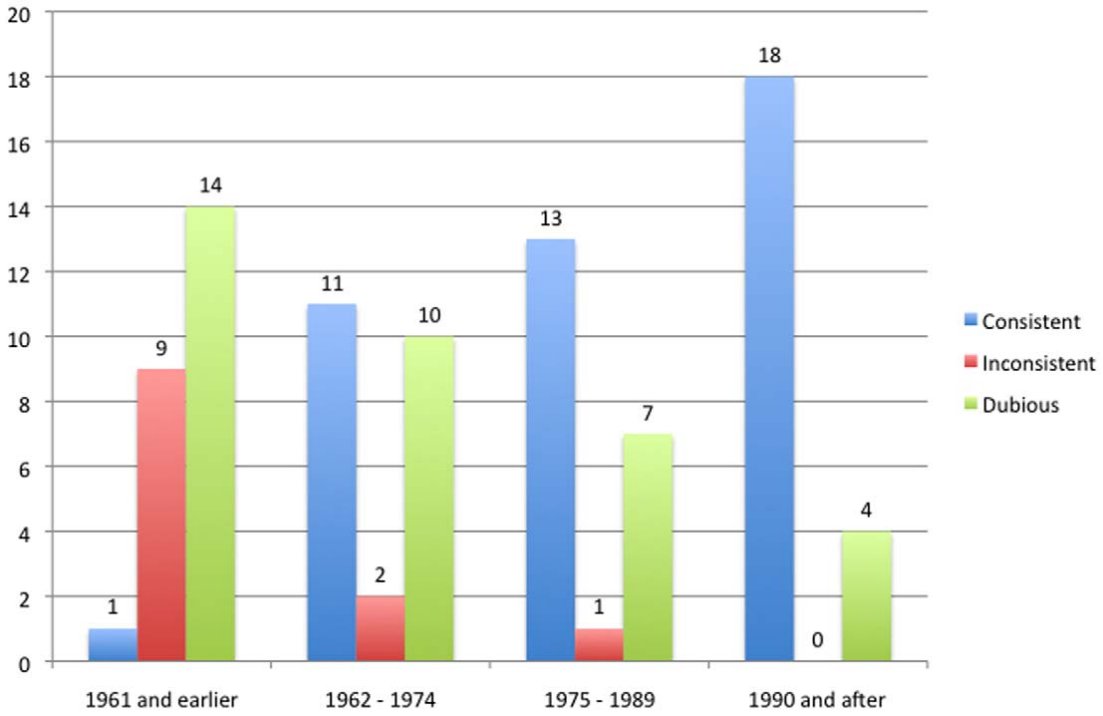
Reliability of study conclusions over time. The figure shows the number of studies in which the conclusion was consistent, i.e., supported by the results (blue bars); inconsistent (red bars), and dubious (green bars), when it depends on a particular interpretation of the data (for instance, post-hoc analysis, multiple outcomes, etc).The number of trials per period was 24 (1961 and earlier), 23 (1962–1974), 22 (1975–1989) and 22 (1990 and after).

We observed that newer trials showed more conclusions consistent with results (when compared to dubious or inconsistent) than older trials (p<0.01), an association that remained significant when the variable “positive or negative results” was inputted in the model (p<0.01). Also, we did not observe a particular trend in more positive results (as compared to negative or unclear results) over time (p = 0.16) (Figures 8, 9 and 10).

Finally, we ran separate analyses for drug class to address whether it could explain the differences observed. Of the 24 analyses performed, we observed associations between the drug class “other” and the variables informed consent (p = 0.01), use of placebo (p = 0.01), randomization (p = 0.02), allocation (p = 0.02), baseline comparison (p = 0.04) and consistency of results (p<0.01); although in all cases the difference was significant only for the group “others” that enrolled fluoxetine and diazepam, not properly showing a “drug class effect”. Also, since the results were only marginally significant, they are probably false-positive findings.

## Discussion

Our results show that the methodology of clinical trials changed substantially over the past 60 years, with significant improvement in quality reporting and in internal validity. The gains in quality reporting were observed in abstract reporting, in which we observed more complete reports in all subsections (background, methods and results) over time. Improvement was also observed in results reporting – as *p* values, effect sizes, baseline group comparisons and adverse effects were more completely reported over time. Also, internal validity increased, since newer studies used more explicit eligibility criteria, objective rating scales, intention-to-treat analyses. Newer studies also showed less biased methods of randomization and blinding. Accordingly, the conclusion of the results of newer studies were more appropriate and consistent than older trials. Study design also changed in some aspects over time: sample size increased, more studies performed (or reported) sample size calculations, and 2-arm substituted 3-or-more-arm designs over time. Placebo use did not change. We further discuss some topics in which these changes impacted the development of clinical trials and discuss future directions based on these results.

First, some limitations should be addressed. One issue is that we based our results on the reports; therefore it is possible that some methodological flaws we encountered were due to lack of reporting. Also, publication bias was a potential issue in our study as we limited our study to articles published only in high-standard journals.

We observed that the quality of abstract reporting improved over the past 60 years. One possible explanation is that journal editors and clinical researchers had noticed that reports of statistics, randomization and baseline comparisons were poor [44], [45] and proposed a set of guidelines to improve the reporting of clinical trials, which ultimately led to the CONSORT statement [46]. However, our results showed that abstract reporting improved significantly *before* CONSORT; on the other hand, recent reviews [47], [48] of abstract reporting in top impact-factor journals showed improvement also *after* CONSORT and also that many top journals had not been referring to CONSORT or alternative abstract guidelines, or had referred to old CONSORT versions. Thus, another possible reason for this improvement is that the abstract gained more importance recently as it is openly available in web databases, becoming an essential piece of information to decide whether or not the full manuscript should be read. In fact, frequently, only the abstract is read, thus supporting its conciseness showing the main characteristics of study design (the reader should understand how the main hypothesis was tested by reading the abstract), main results presented as clearly and simply as possible and future implications of findings, avoiding overstatements.

Moreover, more trials reported the eligibility criteria used, confirmed the diagnostics with structured interviews rather than clinical evaluation and used severity rating scales rather than personal judgment on improvement. Using structured questionnaires improves study validity and reliability – as they are more sensitive to perform differential diagnoses [49] and have more agreement between raters than unstructured evaluations [50]. Reporting the eligibility criteria and using severity rating scales allows readers and researchers to assess the targeted sample and thus to evaluate the generalizability of the study results [51]. However, diagnostic criteria standardization can also generate heterogeneous diagnostic groups. For instance, according to DSM-IV criteria, there are 93 different combinations of depressive symptoms [6], reflecting patients with different characteristics that are in the same “depression DSM-IV” classification.

Severity rating scales also increase internal validity by addressing drug efficacy either quantitatively (score reduction) or qualitatively (response and remission rates). These rating scales are also useful to screen and recruit patients, assess severity, define predictors of response [52] and importantly, to compare the results across different studies. Thus, psychometric scales grant more precision when measuring outcomes. On the other hand, they require proper training to gain satisfactory inter-rater reliability [53] and also are limited. An example of its limitation can be seen through the Hamilton Depression Rating Scale. This scale is excessively weighted in anxiety and somatic symptoms but has little coverage for important depression symptoms [7]. Therefore, although diagnostic standardization certainly increased internal validity, there is still a significant margin for more diagnostic refinement.

Sample size increased over time, however this was marginally significant and could be explained by one large trial with a very large sample [28]. However, the number of multicentric studies also increased, perhaps explaining this finding. In addition, more trials performed (or reported) sample size calculations, which can be explained by several reasons, such as: (1) ethical and economical issues in enrolling more subjects than necessary for the primary hypothesis; (2) statistical improvement over time, allowing a more precise estimation of sample size; (3) increase in scientific rigor over time, as researchers are demanded to state their primary hypothesis *a priori*; (4) concern with negative results due to lack of statistical power.

Regarding study design, we observed that recent trials favor two-arm design while old trials favor three-arm and other designs. Possible reasons are: (1) less prior knowledge on drug effects (e.g., carry-over effects); (2) sponsorship interest of pharmaceutical companies on researching a specific drug and; (3) scarce use of meta-analytic techniques that favor two-arm studies in the past. In addition, we observed that newer trials performed more intention-to-treat analysis, a method used to handle with differential dropouts in treatment groups, increasing the internal validity of the study [54].

Placebo use did not change and remained elevated over time. Although a full review on placebo is beyond our scope, two aspects are important: the ethical issues when considering the use of informed consent and the statistical/methodological importance of placebo in clinical trials. In 1970, Baastrup et al. [30] argued they would not inform patients that lithium would be changed to placebo because there was still uncertainty on its prophylactic effects. The lack of the principle of autonomy can be seen in which the patients themselves have the right to decide whether or not is in their best interest to, for instance, stop taking a given drug. Another important issue is that placebo response in comparison to the active group has increased over time [55], which could theoretically reflect an improvement in internal validity, as robust studies are less susceptible to accidentally breaking blinding. Nevertheless, some reasons explaining the past and present elevated placebo use include: it maximizes assay sensitivity of a trial; therefore amplifying the signal [56]; placebo-controlled studies need smaller sample sizes [13] and the relatively low risk of using placebo in psychiatric trials for short periods of time [57].

Regarding statistics, we observed that more trials reported *p* values over time. This trend was also observed in a review of statistical methods in rehabilitation literature [58], probably reflecting more rigor in data reporting as well as more training in clinical research. In fact, perhaps “forcing” the authors (through structured reporting guidelines) to report *p* values contributed to increase their understanding of statistical methods. This is an important issue when the statistics is done by a third party statistician. Also, we observed newer trials using more parametric tests for the primary hypothesis. Parametric tests increase study efficiency, as such tests are more powerful and outcomes are expressed in score changes rather than response/relapse rates; therefore decreasing sample size requirements. However there is a concern whether it is appropriate using parametric tests for psychiatric rating scales, which are constructed by several items whose range of symptoms assessed are not continuous, but ordinal (e.g., questions about weight loss are usually divided in less than 0.5kg; between 0.5-1kg; more than 1kg).

Randomization techniques also improved over time; however the overall number of adequate reporting was quite low, even for newer trials. This is surprising, as inadequate methods of randomization and allocation are considered major sources of bias [59], [60]. However, here there is the issue of trial quality vs. reporting quality that is highly debated in the literature. For instance, Devereaux et al. [61] contacted authors from 98 randomized controlled trials published after 1997 that failed to report one or more of the RCT procedures. By asking the authors of these trials, Deveraux et al. found that although many trials failed to report some aspects of trial designs, the procedures were indeed performed in almost all studies. On the other hand, Liberati et al. [62] reviewed 119 trials published from 1963 to 1986 and concluded that the overall low methodological quality of the trials (assessed through a score system) only mildly improved after a re-checking with the authors; and Schulz et al. [63], assessed trial quality in 250 RCTs; and found that poor quality is related to bias. In addition, there is no method of choice in assessing bias and trial quality [64].

Along these lines, we verified several aspects of study design (baseline group comparisons; adverse effects reporting; dropout reasons, type of statistical test used) to assess whether the conclusions presented were consistent. Studies rated as “inconsistent” were of quite low quality, while “consistent” studies had good quality. Almost one third of the studies were rated of “dubious” quality in which we did not draw definite conclusions due to incomplete reporting or tendentious data interpretation. Because of that, we think that an important aim for manuscript publication is to allow different researchers to replicate and thus to test the results of the studies. This would allow readers to critically interpret these studies. In order to do so, the authors must detail carefully the methods of their experiments [65]. Also, there is no reason to not fully report all aspects of the study design, particularly at the present time when journal editors and reviewers use structured checklists to assess complete reporting and the authors are able to address missing points when reviewing their papers. Finally the issue of space can always be resolved with supplementary online publication (even pointing out the methods section to a webpage with detailed methodology is now possible). Importantly, our results show that newer trials reported more conclusions in line with the results, thus reflecting gains in reporting *and* quality.

### Conclusion

The psychopharmacological revolution that has been observed since 1949 brought significant challenges for psychiatric research, a field that virtually lacked drug treatment at that time. Some changes include the adoption of operational diagnostic criteria and psychometrics as well as assimilation of novel breakthrough methods of clinical trial research. As a result, clinical trial quality of psychopharmacological studies has changed significantly during the past 60 years in several aspects such as study design, sampling, randomization, allocation, statistical methods, ethical aspects and reporting. In fact, only the use of placebo remained stable in this period. These changes have increased study efficiency and internal validity by systematically detecting, addressing and eliminating various sorts of bias. However, there is room further improvement in the development of rating scales and more refined diagnostic criteria as well as better reporting of some aspects of trial methodology. Therefore, despite the significant advancements observed with better designed and more reliable trials as compared to the past, it is still uncertain that we have achieved the optimal clinical trials methodology.

## Supporting Information

Table S1 QUOROM Checklist.
(0.03 MB DOC)

Table S2 Table S2 shows the main characteristics of each study - the drug studied, the name of the author, the year and the journal published; the disorder analyzed; the study design, the use of wash-out, run-in, intention-to-treat (ITT) periods and informed consent; the sample size (SS) estimation and the report of the primary hypothesis; the description of methods of randomization, allocation and blinding; the number of patients enrolled (n) and the duration of the trial; the reporting of baseline comparisons between groups, drug adverse effects (AE) and reasons for drop-outs (DO) and, finally; the reporting of p values, score values and effect size (ES) estimation. Chlor = chlorpromazine; Li = lithium; D = diazepam; Cloz = clozapine; Flu = fluoxetine; Risp = risperidone; Lam = lamotrigine; BJP = The British Journal of Psychiatry; BMJ = The British Medical Journal; AJP = The American Journal of Psychiatry; Arch = The Archives of General Psychiatry; JCP = The Journal of Clinical Psychiatry; MDD = major depressive disorder; OCD = obsessive-compulsive disorder; MDI = manic-depressive illness; CO = cross-over.
(0.40 MB DOC)
