# Supplementary material for: Changes in Clinical Trials Methodology Over Time: A Systematic Review of Six Decades of Research in Psychopharmacology
Source: PLoS One. 2010 Mar 3;5(3):e9479. doi: 10.1371/journal.pone.0009479 (PMC2831060; doi:10.1371/journal.pone.0009479)
Supplement: Table S2 — shows the main characteristics of each study - the drug studied, the name of the author, the year and the journal published; the disorder analyzed; the study design, the use of wash-out, run-in, intention-to-treat (ITT) periods and informed consent; the sample size (SS) estimation and the report of the primary hypothesis; the description of methods of randomization, allocation and blinding; the number of patients enrolled (n) and the duration of the trial; the reporting of baseline comparisons between groups, drug adverse effects (AE) and reasons for drop-outs (DO) and, finally; the reporting of p values, score values and effect size (ES) estimation. Chlor = chlorpromazine; Li = lithium; D = diazepam; Cloz = clozapine; Flu = fluoxetine; Risp = risperidone; Lam = lamotrigine; BJP = The British Journal of Psychiatry; BMJ = The British Medical Journal; AJP = The American Journal of Psychiatry; Arch = The Archives of General Psychiatry; JCP = The Journal of Clinical Psychiatry; MDD = major depressive disorder; OCD = obsessive-compulsive disorder; MDI = manic-depressive illness; CO = cross-over. (0.40 MB DOC) [file pone.0009479.s002.doc]

**Table 2** shows the main characteristics of each study – the drug studied, the name of the author, the year and the journal published; the disorder analyzed; the study design, the use of wash-out, run-in, intention-to-treat (ITT) periods and informed consent; the sample size (SS) estimation and the report of the primary hypothesis; the description of methods of randomization, allocation and blinding; the number of patients enrolled (n) and the duration of the trial; the reporting of baseline comparisons between groups, drug adverse effects (AE) and reasons for drop-outs (DO) and, finally; the reporting of *p* values, score values and effect size (ES) estimation. Chlor= chlorpromazine; Li= lithium; D= diazepam; Cloz= clozapine; Flu= fluoxetine; Risp= risperidone; Lam=lamotrigine; BJP = The British Journal of Psychiatry; BMJ = The British Medical Journal; AJP = The American Journal of Psychiatry; Arch= The Archives of General Psychiatry; JCP = The Journal of Clinical Psychiatry; MDD= major depressive disorder; OCD= obsessive-compulsive disorder; MDI= manic-depressive illness; CO=cross-over.

| **Drug** | **Author, Year, Journal** | **Disorder** | **Design** | **Washout/ Runin / ITT / Inf Consent** | **SS/ Prim Hyp** | **Random/Alloc/ Blind** | **n / trial duration** | **Baseline / AE / DO** | **p-value / Score / ES** |
| --- | --- | --- | --- | --- | --- | --- | --- | --- | --- |
|
| **Chlor** | Rees, 1955, BJP [64] | Anxiety disorders | CO | N/N/N/N | N/N | N/N/ Y | 150/ 60 | I / I / N | I / N / N |
| **Chlor** | Boardman, 1956,Lancet [31] | Schizophrenia | 2-arm, parallel | Drug-free/N/N/N | N/N | Y/Y/Y | 100/84 | Y/N/Y | Y/Y/N |
| **Chlor** | Lomas, 1957, BMJ [32] | Psychosis | 2-arm, parallel | N/N/N/N | N/N | Y/Y/Y | 100/180 | I/I/Y | N/N/N |
| **Chlor** | Salisbury, 1957, BJP [65] | Schizophrenia | CO | N/Y/N/N | N/N | N/N/Y | 48/140 | I/I/Y | N/N/N |
| **Chlor** | Good, 1958, AJP [66] | Schizophrenia | CO | N/N/N/N | N/N | N/N/Y | 112/180 | N/N/N | Y/Y/N |
| **Chlor** | Smith 1958, AJP [67] | Schizophrenia | Other | N/N/N/N | N/N | N/N/ Y | 85/90 | N/N/N | N/N/N |
| **Chlor** | Fink, 1958, JAMA [33] | Psychosis | 2-arm, parallel | N/N/N/N | N/N | Y/Y/Y | 60/90 | I/Y/N | N/N/N |
| **Chlor** | Dransfield, 1958, BJP [26] | Schizophrenia | 2-arm, parallel | N/Y/N/N | N/N | N/N/ Y | 50/59 | Y/Y/N | N/N/N |
| **Chlor** | Fleming, 1958, BJP [68] | Schizophrenia | Other | N/N/N/N | N/N | N/N/ Y | 36/84 | N/N/Y | Y/Y/N |
| **Chlor** | Foote, 1958, BJP [34] | Psychosis | Other | N/N/N/N | N/N | Y/Y/Y | 70/140 | N/N/Y | N/N/N |
| **Chlor** | Little, 1958, BJP [69] | several diseases | Other | N/N/N/N | N/N | N/N/ Y | 142 | I/I/Y | I/N/N |
| **Chlor** | King, 1958, AJP [37] | Schizophrenia | CO | Drug-free/N/N/N | N/N | Y/Y/Y | 100/180 | I/I/Y | I/N/N |
| **Chlor** | Robinson, 1959, Arch [43] | Elderly Psychotic | 3-arm,parallel | N/Y/N/N | N/N | N/N/ Y | 80/42 | I/I/I | N/N/N |
| **Chlor** | Fleming, 1959, BJP [70] | Schizophrenia | 3-arm,parallel | Y/N/N/N | N/N | N/N/ Y | 63/51 | I/I/Y | I/Y/N |
| **Chlor** | Walsh, 1959, BJP [71] | Schizophrenia | Other | N/N/N/N | N/Y | N/N/ Y | 66/56 | Y/I/Y | I/Y/N |
| **Chlor** | King, 1959, AJP [72] | Schizophrenia | 3-arm,parallel | N/N/N/N | N/N | N/N/ Y | 72/70 | I/I/N | I / N / N |
| **Chlor** | Gilmore, 1959, BJP [73] | Schizophrenia | 3-arm,parallel | N/N/N/N | N/N | N/N/ Y | 45/21 | I/N/Y | Y/Y/N |
| **Chlor** | Hamilton, 1960, BJP [38] | Schizophrenia | Other | Y/N/N/N | N/N | N/N/ Y | 54/56 | N/N/N | I/Y/N |
| **Chlor** | Hurst, 1960, BJP [74] | Paraphrenia | Other | Y/Y/N/N | N/N | N/N/ Y | 92/56 | Y/Y/Y | Y/Y/N |
| **Chlor** | Abse, 1960, JAMA [39] | Elderly Psychotic | 3-arm,parallel | N/N/N/N | N/N | N/N/ Y | 129/56 | Y/I/N | N/N/N |
| **Chlor** | Casey, 1960, AJP [75] | Schizophrenia | 3-arm,parallel | Drug-free/N/N/N | N/N | N/N/ Y | 640/84 | I/Y/Y | I/N/N |
| **Chlor** | Casey, 1961, AJP [76] | Schizophrenia | Other | N/N/N/N | N/N | N/N/ Y | 520/140 | I/I/Y | I/N/N |
| **Chlor** | Aschroft, 1961, BJP [77] | Schizophrenia | Other | Y/Y/N/N | N/N | N/N/ Y | 52/140 | I/Y/Y | I / N / N |
| **Chlor** | Wilson, 1961, BJP [78] | Schizophrenia | Other | N/Y/N/N | N/N | N/N/ Y | 8/56 | I/I/Y | Y/Y/N |
| **Li** | Maggs, 1963, BJP [79] | Mania | CO | Drug-free/N/N/N | N/N | N/N/ Y | 28/42 | Y/Y/Y | I/N/N |
| **D** | Capstick, 1965, BJP [80] | Anxiety States | Other | N/N/N/N | N/N | N/N/ Y | 30/28 | N/I/Y | I/N/N |
| **D** | McDowall, 1966, BJP [81] | Anxiety States | CO | N/N/N/N | N/N | N/N/ Y | 28/28 | I/Y/Y | Y/Y/N |
| **D** | Nesselhof, 1966, AJP | Anxiety States | 3-arm,parallel | N/N/N/N | N/N | N/N/ Y | 42/42 | Y/I/N | I/Y/N |
| **Li** | Fieve, 1968, AJP [15] | Bipolar / MDI | 2-arm, parallel | N/Y/N/N | N/N | N/N/ Y | 29/21 | I/N/N | N/N/N |
| **Li** | Melia, 1970, BJP [82] | Affective disorders | 2-arm, parallel | N/Y/Y/N | N/N | N/N/ Y | 18/720 | Y/I/Y | I/N/N |
| **Li** | Spring, 1970, AJP [83] | Mania | 2-arm, parallel | Drug-free/N/N/N | N/N | Y/N/Y | 14/21 | N/Y/N | N/N/N |
| **Li** | Platman, 1970, AJP [42] | Mania | 2-arm, parallel | Drug-free/N/N/N | N/N | N/N/ Y | 23 | Y/N/Y | N/N/N |
| **Li** | Baastrup, 1970 Lancet [30] | Bipolar / MDI | 2-arm, parallel | N/Y/N/N | Y/Y | N/N/ Y | 91/150 | Y/I/Y | Y/Y/N |
| **D** | Kay, 1970, BJP [27] | Affective disorders | 2-arm, parallel | N/N/N/N | N/N | N/N/ Y | 132/210 | I/I/Y | Y/Y/N |
| **Li** | Stokes, 1971, Lancet [84] | Mania | CO | N/N/N/N | N/N | N/N/ Y | 38 | I/N/N | I/N/N |
| **Li** | Coppen, 1971, Lancet [85] | Affective disorders | 2-arm, parallel | N/N/N/N | N/N | N/N/ Y | 65/700 | Y/N/Y | Y/Y/N |
| **Li** | Johnson, 1971, BJP [86] | Mania | 2-arm, parallel | Y/Y/N/Y | N/N | N/N/ Y | 34/21 | I/I/Y | I/Y/N |
| **D** | Haider, 1971,BJP [25] | Anxiety States | 2-arm, parallel | N/N/N/N | N/N | N/N/ Y | 50/21 | I/N/Y | I/Y/N |
| **D** | Holliste, 1971, Arch [41] | Anxiety States | 2-arm, parallel | N/N/N/N | N/N | Y/N/Y | 67/28 | N/I/N | I/Y/N |
| **Li** | Prien, 1972, Arch [87] | Mania | 2-arm, parallel | Drug-free/N/N/N | N/N | N/N/ Y | 255/21 | I/Y/Y | I/N/N |
| **D** | Wadzisz, 1972, BJP [88] | Anxiety States | 2-arm, parallel | N/N/N/N | N/N | N/N/ Y | 40/28 | I/N/N | I/N/N |
| **D** | Marks, 1972, BJP [89] | Anxiety States | CO | N/N/N/Y | N/N | N/N/ Y | 18/1 | I/N/N | Y/N/N |
| **Li** | Prien, 1973, Arch [90] | Bipolar / MDI | 2-arm, parallel | N/Y/N/N | N/Y | N/N/ Y | 205/720 | I/N/Y | Y/Y/N |
| **Li** | Naylor, 1974, BJP [91] | Affective disorders | CO | N/N/N/N | N/N | N/N/ Y | 17/700 | Y/I/Y | Y/N/N |
| **Cloz** | Shopsin, 1974, Arch [92] | Bipolar / MDI | 2-arm, parallel | Y/Y/N/N | N/N | N/N/ Y | 30/21 | Y/I/Y | I / N / N |
| **Li** | Prien1, 1974, AJP [93] | Affective disorders | 2-arm, parallel | N/N/N/N | N/Y | N/N/ Y | 205/720 | Y/I/N | I / N / N |
| **Li** | Prien2, 1974, AJP [90] | Affective disorders | 3-arm,parallel | N/N/N/N | N/Y | N/N/ Y | 122/720 | Y/I/N | I/N/N |
| **Li** | Fieve, 1975, Arch [94] | MDD, Unipolar | 2-arm, parallel | N/N/N/N | N/N | N/N/ Y | 52/1460 | Y/I/Y | Y/Y/N |
| **Li** | Takahashi, 1975, Arch [95] | Mania | 2-arm, parallel | Y/N/N/N | N/N | N/N/ Y | 80 | I/I/Y | I/Y/N |
| **Cloz** | Van Praag, 1976, BJP [96] | Psychosis | 2-arm, parallel | Y/N/N//N | N/N | N/N/ Y | 28/10 | I/I/Y | I/N/N |
| **Li** | Coppen, 1976, BJP [97] | Affective disorders | 2-arm, parallel | N/N/N/N | N/N | N/N/ Y | 23/360 | Y/I/Y | Y/N/N |
| **Li** | Dunner, 1976, Arch [98] | Bipolar / MDI | 2-arm, parallel | Y/N/N/N | N/N | N/N/ Y | 40/480 | Y/N/Y | Y/ N / N |
| **Li** | Watanabe, 1976, Arch [99] | MDD, Unipolar | 2-arm, parallel | Y/N/N/N | N/N | N/Y/Y | 64/35 | Y/Y/Y | I/N/N |
| **Cloz** | Gelenberg, 1979, JCP [100] | Schizophrenia | 2-arm, parallel | Y/N/N/N | N/N | N/N/ Y | 15/42 | I/I/Y | N / N / N |
| **Cloz** | Shopsin, 1979, Arch [101] | Bipolar / MDI | 3-arm,parallel | Y/Y/N/Y | N/N | N/N/ Y | 31/35 | I/I/I | N/N/N |
| **Flu** | Bremner, 1984, JCP [102] | MDD, Unipolar | 2-arm, parallel | Drug free/Y/N/Y | N/N | N/N/ Y | 40/35 | I/Y/Y | Y/Y/N |
| **Flu** | Chouinard, 1985, JCP [103] | MDD, Unipolar | 2-arm, parallel | Y/Y/N/Y | N/N | N/N/Y | 51/35 | Y/Y/Y | Y/Y/N |
| **Flu** | Cohn, 1985, JCP [104] | MDD, Unipolar | 2-arm, parallel | Y/Y/Y/Y | N/N | N/N/Y | 166/42 | Y/Y/N | Y/N/Y |
| **Flu** | Rickels, 1985, JCP [105] | MDD, Unipolar | 2-arm, parallel | Drug free/Y /Y/Y | N/N | N/N/Y | 185/42 | I/I/N | N/N/N |
| **Flu** | Feighner1, 1985, JCP [106] | MDD, Unipolar | 2-arm, parallel | Y/Y/Y/Y | N/N | N/N/Y | 157/42 | Y/Y/Y | N/N/N |
| **Flu** | Feighner2, 1985, JCP [107] | MDD, Unipolar | 2-arm, parallel | Drug free/Y/Y/N | N/N | N/N/ Y | 44/35 | I/Y/N | Y / N / N |
| **Flu** | Fabre, 1987, JCP [108] | MDD, Unipolar | 2-arm, parallel | Drug free/Y/N/Y | N/Y | N/N/ Y | 84/42 | I/I/N | I / N / N |
| **Flu** | Young, 1987, BJP [109] | MDD, Unipolar | 2-arm, parallel | Drug free/N/N/N | N/N | N/N/ Y | 64/42 | I/Y/Y | I/I/N |
| **Flu** | Levine, 1987, BJP [110] | MDD, Unipolar | 2-arm, parallel | Y/N/N/Y | N/N | N/N/ Y | 60/42 | Y/I/N | I/I/N |
| **Cloz** | Kane, 1988, Arch [111] | Schizophrenia | 2-arm, parallel | Y/Y /Y/Y | N/Y | N/N/ Y | 268/42 | Y/Y/Y | Y/N/N |
| **Flu** | Debus, 1988, JCP [112] | MDD, Unipolar | 2-arm, parallel | Drug free/Y/N/Y | N/N | N/N/ Y | 43/42 | Y/Y/Y | Y/Y/N |
| **Flu** | Laakmann, 1988, BJP [113] | MDD, Unipolar | 2-arm, parallel | Y/Y/N/Y | N/N | N/N/ Y | 130/35 | I/N/N | I/Y/N |
| **Flu** | Montgomery, 1988, BJP [114] | MDD, Unipolar | 2-arm, parallel | N/Y /N/N | N/Y | N/N/ Y | 220/360 | I/N/N | I/Y/N |
| **Flu** | Perry, 1989, JCP [115] | MDD, Unipolar | 2-arm, parallel | Drug free/Y/N/N | N/N | N/N/ Y | 40/42 | Y/Y/Y | I/N/N |
| **Flu** | Pigott, 1990, Arch [116] | OCD | CO | Y/Y /Y/N | N/N | N/N/ Y | 11/168 | Y/Y/Y | Y/Y/N |
| **Flu** | Usher, 1991, JCP [117] | MDD, Unipolar | 2-arm, parallel | Drug free/Y/Y/ N | N/N | N/N/ Y | 120/42 | Y/Y/Y | I/N/N |
| **Flu** | Feighner, 1991, JCP [118] | MDD, Unipolar | 2-arm, parallel | Drug free/Y/Y/Y | N/N | N/N/ Y | 137/42 | Y/Y/Y | I/Y/N |
| **Cloz** | Pickar, 1992, Arch [119] | Schizophrenia | Other | Y/Y /Y/Y | N/Y | N/N/ Y | 21/100 | Y/Y/Y | Y/Y/N |
| **Cloz** | Breier, 1994, AJP [120] | Schizophrenia | 2-arm, parallel | Y/Y /N/N | N/Y | Y/Y/Y | 45/70 | Y/Y/Y | Y/Y/N |
| **Risp** | Marder, 1994, AJP [121] | Schizophrenia | 2-arm, parallel | Y/N/Y/Y | N/Y | Y/Y/Y | 388/56 | Y/Y/Y | Y/Y/N |
| **Risp** | Peuskens, 1995, JCP [28] | Schizophrenia | 2-arm, parallel | Y/Y PL/Y/Y | Y/Y | Y/Y/Y | 1362/56 | Y/Y/Y | Y/Y/Y |
| **Risp** | Bondolfi, 1998, AJP [122] | Schizophrenia | 2-arm, parallel | Y/N/Y/Y | N/Y | N/N/ Y | 86/56 | Y/Y/Y | Y/Y/Y |
| **Risp** | Lindenmayer, 1998, JCP [35] | Schizophrenia | 2-arm, parallel | Y/N/Y/Y | N/N | Y/Y/Y | 35/84 | Y/Y/Y | Y/Y/N |
| **Riso** | Wirshing, 1999, AJP [123] | Schizophrenia | 2-arm, parallel | Y/Y /N/Y | N/N | Y/N/Y | 67/28 | Y/Y/Y | Y/Y/N |
| **Risp** | Breier, 1999, AJP [124] | Schizophrenia | 2-arm, parallel | Y/Y /N/N | N/N | N/N/ Y | 29/42 | Y/Y/Y | Y/Y/Y |
| **Risp** | Katz, 1999, JPC [125] | Schizophrenia | 2-arm, parallel | Y/N/Y/Y | Y/Y | Y/N/Y | 625/84 | Y/Y/Y | Y/N/N |
| **Risp** | Ho, 1999, JCP [36] | Schizophrenia | 2-arm, parallel | Drug free/N/N/N | N/N | Y/Y/Y | 42/180 | Y/I/N | Y/Y/N |
| **Lam** | Calabrese, 1999, JCP [126] | Bipolar / MDI | 2-arm, parallel | Y/N/Y/Y | Y/Y | N/N/ Y | 195/49 | Y/Y/Y | I/N/N |
| **Risp** | McDougle, 2000, Arch [24] | OCD | 2-arm, parallel | N/N/N/Y | N/Y | Y/N/Y | 36/42 | Y/Y/Y | Y/Y/N |
| **Lam** | Calabrese, 2000, JCP [127] | Bipolar / MDI | 2-arm, parallel | Y/Y /Y/Y | N/Y | N/N/ Y | 182/182 | Y/Y/Y | Y/N/N |
| **Risp** | Azorin, 2001, AJP [128] | Schizophrenia | 2-arm, parallel | Y/Y / N /Y | Y/Y | Y/N/Y | 256/84 | Y/Y/Y | Y/Y/N |
| **Risp** | Conley, 2001, AJP [129] | Schizophrenia | 2-arm, parallel | Y/N/Y/Y | N/N | N/N/ Y | 377/56 | Y/Y/N | Y/Y/Y |
| **Lam** | Normann, 2002, JCP [130] | Bipolar / MDI | 2-arm, parallel | Drug free/Y /Y/Y | N/Y | Y/N/Y | 40/63 | Y/Y/Y | I/N/N |
| **Lam** | Bowden, 2003, Arch [131] | Bipolar / MDI | 3-arm,parallel | Y/Y /Y/Y | Y/Y | N/N/ Y | 175/490 | Y/Y/Y | Y/Y/Y |
| **Lam** | Calabrese, 2003, JCP [132] | Bipolar / MDI | 3-arm,parallel | Y/Y /Y/Y | Y/Y | N/N/ Y | 463/490 | Y/Y/Y | Y/Y/Y |
| **Lam** | Barbosa, 2003, JCP [40] | Bipolar / MDI | 2-arm, parallel | Y/N/Y/Y | N/N | Y/N/Y | 23/42 | I/I/Y | Y/N/N |
